# Sphingolipid Metabolism Perturbations in Rett Syndrome

**DOI:** 10.3390/metabo9100221

**Published:** 2019-10-10

**Authors:** Gerarda Cappuccio, Taraka Donti, Michele Pinelli, Pia Bernardo, Carmela Bravaccio, Sarah H. Elsea, Nicola Brunetti-Pierri

**Affiliations:** 1 Department of Translational Medicine, Federico II University, 80131 Naples, Italy; g.cappuccio@tigem.it (G.C.); m.pinelli@tigem.it (M.P.); pia.bernardo84@gmail.com (P.B.); carmela.bravaccio@unina.it (C.B.); 2 Telethon Institute of Genetics and Medicine, Pozzuoli, 80078 Naples, Italy; 3 Department of Molecular and Human Genetics, Baylor College of Medicine, Houston, TX 77030, USA; tarakdonti@gmail.com

**Keywords:** Rett syndrome, sphingolipid, sphinganine, sphingosine, MECP2

## Abstract

Rett syndrome is a severe neurodevelopmental disorder affecting mostly females and is caused by loss-of-function mutations in the *MECP2* gene that encoded the methyl-CpG-binding protein 2. The pathogenetic mechanisms of Rett syndrome are not completely understood and metabolic derangements are emerging as features of Rett syndrome. We performed a semi-quantitative tandem mass spectrometry-based analysis that measured over 900 metabolites on blood samples from 14 female subjects with Rett syndrome carrying *MECP2* mutations. The metabolic profiling revealed alterations in lipids, mostly involved in sphingolipid metabolism, and sphinganine/sphingosine, that are known to have a neurotrophic role. Further investigations are required to understand the mechanisms underlying such perturbations and their significance in the disease pathogenesis. Nevertheless, these metabolites are attractive for studies on the disease pathogenesis and as potential disease biomarkers.

## 1. Introduction

Unbiased metabolic analyses can be used for the diagnosis of inborn errors of metabolism, but are also emerging as a powerful tool to detect metabolic perturbations involved in disease pathogenesis [1,2,3]. Rett syndrome is a severe neurodevelopmental disorder largely affecting females, with an incidence of 1:10,000 [4,5]. It is caused by loss-of-function mutations in the gene encoding methyl-CpG-binding protein 2 (*MECP2*) on the X chromosome [6]. Typically, in the first 6–18 months of life, girls with Rett syndrome have an apparently normal development that is followed by loss of acquired skills, seizures, and intellectual disability [7]. Symptoms progress over stages of developmental stagnation, rapid regression, plateau, and late motor deterioration. The stagnation stage is characterized by delays in motor and language skills, and is followed by rapid regression with loss of purposeful hand skills and spoken language, motor impairments, breathing abnormalities, and seizures. This period is then followed by a plateau stage and a late motor deterioration. However, clinical presentations may vary widely, and this variability likely depends on the type of mutation, X chromosome inactivation, and modifier genes. Current treatments for Rett syndrome are only symptomatic [4] but several treatments, including gene therapy, are under preclinical investigation and might become available in the near future [8].

Studies in a Rett syndrome mouse model detected metabolic alterations, mostly involving cholesterol metabolism [9]. However, to our knowledge there have been no studies on human samples aiming at investigating the metabolic derangements of Rett syndrome. In the present pilot study, we used an ultra-high performance liquid chromatography-tandem mass spectrometry to detect metabolic alterations in plasma samples of 14 Rett syndrome subjects. This semi-quantitative, tandem mass spectrometry-based method allows a comprehensive evaluation of over 900 metabolites (amino acids, organic acids, fatty acids, neurotransmitters, nucleotides, cofactors and vitamins, bile acids, and other molecules < 1000 Da in molecular weight) [1]. 

## 2. Subjects and Methods

### 2.1. Participants and Clinical Data

The study was approved by the Ethics Committee of Federico II University Hospital in Naples, Italy (protocol number 51/16, approved on April 2016), and was performed in accordance to the World Medical Association Declaration of Helsinki. Female subjects (n = 14 with an age range of 3 to 29 years and a mean age of 14.4 years) with Rett syndrome confirmed by *MECP2* mutations were enrolled in the study. Enrolled subjects were regularly followed up with at the child neuropsychiatric clinic of Federico II University Hospital. Table 1 summarizes their clinical and genetic data. For each subject, a clinical severity score (range: 1–58) was calculated based on key diagnostic and developmental features of Rett syndrome, such as age at the onset of regression, somatic and head growth, and motor, communication, and Rett syndrome behaviors/other neurologic symptoms [10].

### 2.2. Metabolic Analysis

Metabolic profiling was performed by Baylor Genetics Laboratories (Houston, TX, USA) and Metabolon, Inc. (Durham, NC, USA) as previously described [3,11]. 

### 2.3. Statistical Analyses

Global Metabolomic Assisted Pathway Screen was performed and changes in metabolite levels were expressed as z-scores calculated based on a control population (*n* = 730). The statistical significance of z-score analysis was expressed as false discovery rate (FDR). We used single group t-test to compare the z-score distribution of Rett syndrome subject-group versus controls. In pathway analysis, we evaluated metabolites acting within the same pathway, calculated the mean values for all metabolites belonging to the same pathway, and applied a Kruskal–Wallis analysis to compare mean levels of pathway metabolites versus non-pathway metabolites. Statistical analysis was performed using the R package version 3.3.1. *P*-values < 0.05 were considered as statistically significant. Box plots and scatter plot were designed with ggplot2 R-package and GraphPad Prism7, respectively.

## 3. Results

**Figure 1:**
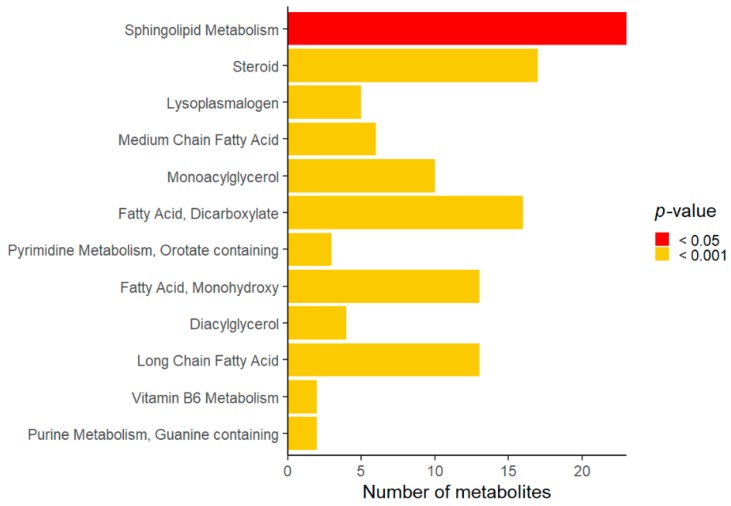
Overview of significant sub-pathways affected in Rett syndrome.

Compared to the controls, Rett syndrome samples showed changes in several metabolites that were analyzed as super-pathways and sub-pathways. Lipid, amino acid, cofactor, and vitamin were the most affected super-pathways (Table 2). Among the lipid super-pathway, sphingolipid, steroid, lyso-plasmalogen, and medium chain fatty acid were significantly different in Rett syndrome subjects compared to the controls (Figure 1).

**Figure 2:**
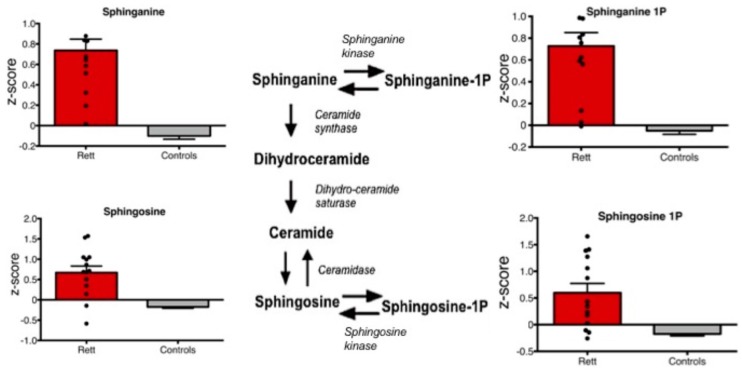
Box plots of plasma levels of specific sphingosine/sphinganine metabolites are shown. All shown metabolites are significantly increased in Rett syndrome cases compared to controls (*p* < 0.01).

Metabolites of the sphinganine/sphingosine pathway including sphinganine, sphinganine 1-phosphate, sphingosine, and sphingosine 1-phosphate were all significantly increased in Rett syndrome subjects compared to controls (*p* < 0.01) (Figure 2). 

## 4. Discussion

The transcription factor *MECP2* regulates expression of a wide range of genes and is emerging as an important regulator of metabolic homeostasis [9,12,13]. Prior to symptom onset, brain cholesterol is increased, and statins were found to ameliorate motor symptoms and to extend lifespan in *Mecp2*-mutant mice [9]. Moreover, *Mecp2*-mutant mice were found to have fatty liver disease, metabolic syndrome, insulin resistance, and changes in energy homeostasis [13]. In addition, mesenchymal stromal cells from a Rett syndrome mouse model showed impaired mitochondrial energy production that was rescued by palmitate-containing media [14]. In Rett syndrome patients, elevated total cholesterol was detected in blood and fibroblasts [15] and a ketogenic diet was found to ameliorate some neurobehavioral symptoms [16,17].

In the present study, we detected significant changes in the lipid super-family, sphingolipid, steroid, lyso-plasmalogen, and medium chain fatty acid sub-pathways in Rett syndrome cases compared to the controls. Moreover, we detected and an increase in sphinganine/sphingosine and their corresponding phosphate forms. Interestingly, a trend towards increased levels of sphinganine, sphingosine, and ceramide was also previously detected in Rett syndrome neurons [18] and increased levels of sphingosine-1P were recently detected in individuals with autism spectrum disorder [19]. The sphingosine pathway plays an important role in signaling and is finely regulated. Depending on its subcellular localization, sphingosine 1-phosphate can regulate mitochondrial function and gene expression by inhibition of histone deacetylases [20]. Moreover, a sphingosine 1-phosphate mimetic, i.e., Fingolimod, was found to improve motor phenotypes and to prolong survival in Rett mouse models [21].

In conclusion, by an untargeted metabolic analysis this pilot study unraveled changes in sphingolipid and sphinganine/sphingosine metabolites in plasma of Rett syndrome subjects. These changes need to be confirmed in a larger cohort of patients, and the mechanisms underlying these perturbations and their consequences in the disease pathogenesis remain to be established. Nevertheless, this study suggests that alterations in sphingolipid metabolism might play a role in Rett syndrome pathogenesis and it may pave the way toward the development of disease-specific biomarkers. 

## Figures and Tables

**Table 1:** Features of subjects included in the study.

Subject	Age (years)	*MECP2* Variant ^a^	Seizures	Drugs	Clinical Severity Score
1	6	c.1116_1201del86	-	Vitamin D	6
2	13	c.455C>G (p.Pro152Arg)	+	Clonazepam, Carbamazepine, Acetazolamide, Lansoprazole, Vitamin D	58
3	12	c.455C>G (p.Pro152Arg)	+	Carbamazepine, Lansoprazole	57
4	10	c.1164_1189del26	-	N-acetyl-cysteine	6
5	16	c.908_1143del236+1159-1170del12	-	Carbamazepine, Insulin	48
6	15	c.763C>T (p.Arg255Ter)	+	Valproate, Lamotrigine, Melatonin	47
7	7	c.1151_1352del202	+	Vitamin D	28
8	11	c.397C>T (p.Arg133Cys)	+	Valproate	36
9	3	c.502C>T (p.Arg168Ter)	-	Carnitine	48
10	17	c.763C>T (p.Arg255Ter)	-	Carbamazepine	43
11	29	c.808C>T (p.Arg270Ter)	-	Carnitine, Carbamazepine, Pentoxifylline, Chlorpromazine	37
12	13	c.502C>T (p.Arg168Ter)	-	Valproate	12
13	21	c.397C>T (p.Arg133Cys)	-	Valproate, Topiramate, Prazosin	12
14	29	c.473C>T (p.Thr158Met)	-	Phenobarbital	16

^a^*MECP2* variants are reported according to NM_004992.3 (NP_004983.1).

**Table 2:** Super-pathways analysis on metabolite perturbations detected in Rett syndrome.

Super-Pathway	Compounds in Super-Pathway
Lipid	305 ***
Amino Acid	147 **
Cofactor and Vitamin	17 *
Xenobiotics	71
Energy	8
Peptide	34
Carbohydrate	22
Nucleotide	28

* *p* < 0.05, ** *p* < 0.01, *** *p* < 0.0005.
